# Association of social capital with self-perceived eHealth literacy among community-dwelling older people: Age and gender differences

**DOI:** 10.3389/fpubh.2023.1088863

**Published:** 2023-04-14

**Authors:** Chenglin Cao, Wenwen Cao, Xin Zheng, Kai Ji, Yunwei Wu, Zhi Hu, Ruoling Chen, Zhongliang Bai

**Affiliations:** ^1^Department of Health Services Management, School of Health Services Management, Anhui Medical University, Hefei, China; ^2^Faculty of Education, Health and Wellbeing, University of Wolverhampton, Wolverhampton, United Kingdom

**Keywords:** healthy aging, social capital, older adults, eHealth literacy, cross-sectional

## Abstract

**Background:**

Studies have confirmed that social factors, including social capital and eHealth literacy, are important in later life. Currently, few studies are available for determining the relationship between social capital and eHealth literacy, and whether such a relationship exists among older people and there are age and gender differences in the relationship remain unclear. Consequently, this study aimed to investigate the association between social capital and eHealth literacy, specifically examing its variations in age and gender.

**Methods:**

A cross-sectional study of 4,257 residents aged ≥ 60 years and dwelling in the community was conducted across four cities in China. A structured questionnaire was used to collect data on general characteristics, socioeconomic status, social capital, and eHealth literacy. Generalized linear models were employed to assess these associations.

**Results:**

There were 4,218 respondents (age 71.9 ± 7.2 years; 64.8% women). Overall, social participation, social connection, trust, cohesion, and reciprocity were all statistically associated with eHealth literacy (*p* < 0.05), while such an association was not observed for social support (*p* > 0.05). Specifically, a higher level of social participation was associated with better eHealth literacy scores among participants aged 70–79 years (*p* < 0.001), and a higher level of social connection was associated with better eHealth literacy scores for those aged 60–69 and 70–79 years (*p* < 0.001). Meanwhile, no gender differences in the associations were found.

**Conclusion:**

There is an association between social capital and eHealth literacy in older men and women. The association varis with age. The findings provide a reference for developing targeted measures to improve self-perceived eHealth literacy among older people. It is essential for achieving active and healthy aging and developing the knowledge and understanding of relevant theories, concepts, and evidence within the field of health and social capital.

## Introduction

The rapid process of population aging, with an increasing demand for medical and health management, has a huge impact on economic and social development worldwide (1). China, as the largest developing country, has also confronted this challenge to satisfy the health needs of older adults and the goal of healthy aging (2).

Recently, an increasing number of studies have identified that social factors are beneficial for health promotion and wellness in old age, among which increasing attention has been paid to social capital (3–5). Social capital is a multi-faceted concept that includes multiple dimensions, each of which describes a phenomenon about social relations at the individual and societal levels and consists of structural social capital and cognitive social capital (6, 7). Structural social capital (social participation, social connection, and social support) usually refers to what is involved in or done through social relationships, emphasizing the concept of action and measuring individual participation in public affairs (8). Cognitive social capital (trust, cohesion, and reciprocity) generally refers to things perceived through ideas, emphasizing concepts at the cognitive level and focusing on the perception of the trustworthiness of social environment (9). More importantly, the significance of social capital in achieving better health outcomes and status has been proven in later life (10, 11). For example, older people with a higher level of social participation and social support are liable to create better self-rated health and functional capabilities (12). The positive effect of some dimensions of social capital on the daily activities of older people has been demonstrated. Likewise, social capital interventions could reduce frailty and promote the development of health-promoting lifestyles among older people (13).

Individuals are increasingly expected to conduct appropriate self-care and self-management through e-medical services and digital devices (14); therefore, eHealth literacy is a dynamic process, defined as the ability of individuals to search, access, understand, evaluate, and use specific health information from electronic sources to make appropriate health decisions (15). eHealth literacy has attracted increasing interest nowadays (16). The relevance of eHealth literacy has been observed in many studies. For example, studies have found that health inequities can be reduced by improving eHealth literacy among socially vulnerable groups (17). During COVID-19 pandemic isolation period, eHealth literacy has been found to provide disease knowledge and publicize and educate on disease prevention behaviors (18, 19). Currently, factors such as advanced age, females, lower educational achievements, and living in remote areas are linked to poor eHealth literacy (20–22).

A previous study confirmed that eHealth literacy mediates the correlation between structural social capital and health behaviors (23). Social capital can be structured *via* not only face-to-face communication but also online connection and virtual socializing (24). In addition, the role of social connection has been observed when there have been challenges in obtaining health information and services from the Internet daily. In other words, individuals will turn to their social relations for help as well as to enhance social interaction and the need for eHealth literacy (25). A previous study also confirmed that a high level of health literacy can enhance the intention to find health information on the Internet (26). Therefore, exploring the relationship between social capital and eHealth literacy has important public health significance.

Moreover, variations in different age ranges and gender groups regarding eHealth literacy have been previously confirmed (27, 28). For example, one study revealed that older people have lower eHealth literacy than their younger counterparts (27). Meanwhile, older females were less likely to know how to find useful health resources on the Internet and have fewer skills to assess health resources, with a relative lack of eHealth literacy (28). Furthermore, when social capital acts positively on health, both age and gender variations have been previously reported (29–31). For instance, a previous study reported that the relationship between social capital and loneliness differs by age group. Specifically, trust was associated with loneliness in all age groups, whereas social participation was associated with loneliness only at younger ages (32). Similarly, social participation was negatively associated with depression in older males only (29). In addition, the relationship between social capital and individual health was found to be influenced by both gender and age, in which a lack of reciprocity was negatively associated with health in males but not in younger females (31).

The relevance of social capital and eHealth literacy in health promotion and education has been widely examined and recognized. However, whether social capital is associated with eHealth literacy requires further exploration. In light of this, in the current study, we associated social capital with eHealth literacy among community-dwelling older people in China, paying special attention to examine age and gender differences in the association. Consequently, these findings will be of great public health significance and help improve eHealth literacy, thus facilitating the process of healthy aging.

## Materials and methods

### Participants and data collection

Between November and December 2020, to recruit eligible participants, we designed cross-sectional research characterized by stratified and multi-stage. Our sampling process can be described as follows:

First, the participants were selected from four cities in the Yangtze River Delta region in China; Jinshan of Shanghai, Huzhou of Zhejiang Province, Changzhou of Jiangsu Province, and Huainan of Anhui Province. Second, each county-level region was randomly selected from four survey areas. After that, from each county-level region, two urban communities (streets) and two rural communities (townships) were randomly selected as urban and rural communities, amounting to 16 communities. Third, we randomly selected 24 communities from above mentioned 16 communities as sampling areas in this study (Additional File 1).

According to the study design, participants aged 60 and older who were absent from deaf/mute or dementia/cognitive impairment and willing to participate in our investigation were eligible for the interview. Before commencing the investigation, we verbally explained the aims and procedures of this study to each participant, after which informed consent was obtained. In total, 4,257 respondents were surveyed and 4,218 (99.08%) were eligible for analysis. The Ethics Committee of Anhui Medical University approved the study protocol (No. 20150297). Details regarding the participants and data collection can be found elsewhere (33).

### Evaluation of the independent variable

Based on our previous studies (3, 4, 34), we used a tool comprising 26 items to assess social capital, the main independent variable. Each dimension was calculated as the sum of its associated items, namely social participation (range 4–20), social support (range 4–20), social connection (range 5–25), trust (range 4–20), cohesion (range 5–25), and reciprocity (range 4–20). Participants who had higher scores suggested a better social capital degree. According to Cronbach’s alpha (Cronbach’s α = 0.870), it can be demonstrated that the internal consistency of this sample is good. The full text of this measurement tool can be found in Additional File 2.

### Evaluation of the dependent variable

This study measured self-perceived eHealth literacy using a simplified five-point Likert Chinese version of eHEALS (35). This scale consists of three dimensions and eight items, including the application ability test of network health information and services (items 1–5), judgment ability test (items 6 and 7), and decision-making ability test (item 8). Participants were asked to choose from 1 to 5, ranging from strongly disagree to strongly agree, respectively. We calculated the eHealth literacy score by adding the scores of the eight items together, with higher scores indicating better eHealth literacy (36). Good internal consistency was also observed in this sample according to the value of Cronbach’s alpha (Cronbach’s α = 0.992). A detailed description of this measurement can be reviewed in Additional File 2.

### Evaluation of related variables

This study included age (years), gender, body mass index (BMI, kg/m^2^), residency (including urban or rural), living status (including living alone or not living alone), marital status (married or single), educational attainment (consisting of primary school or below, junior school, high school, and college or above), smoking status (smoking-quitter, smoker, or non-smoker), and drinking status (drinking-quitter, drinker, or non-drinker). Information on income sources (salary, family provision, subsidy, and others), medical insurance, and endowment insurance were also collected. To compare with previous studies (23, 33, 37, 38), we grouped these variables for data analysis. To achieve statistical power, the participants were divided into three groups.

### Statistical procedure

In the first step, we used mean ± standard deviation and numbers and percentages to express continuous and categorical variables, respectively. In the second step, we explored the linear relationships between independent and dependent variables using Pearson’s r correlation analysis. In the third step, we calculated the standard error and associated 95% confidence interval (95% CI) using generalized linear models (GLM). The fitted GLM can be expressed as follows:


$$\begin{array}{c} Y\approx \alpha +\beta_{1}Socialcapitaldimensions\\ +\beta_{2}Confounders_{1}+\ldots +\beta_{n}Confounders_{n} \end{array}$$

In this equation, Y is the eHealth literacy score, α is the intercept, $\beta_{1}$ is the corresponding coefficient of social capital dimensions, $\beta_{2}\ldots \beta_{n}$ indicates the coefficients of the covariates included in the regression models. During the data analysis, we checked and excluded collinearity among the analyzed variables according to the variance inflation factor (VIF) results (VIF greater than 10 indicates the presence of collinearity). In this study, SPSS 22.0 was used for all data analyses, and statistical significance was set at *p* < 0.05.

## Results

### General information on participants

As revealed in Table 1, 4,218 participants (age 71.9 ± 7.2 years), namely 1,484 males (35.2%) and 2,734 females (64.8%), were included. Of them, rural residents accounted for 45.1, and 86.5% of the participants did not live alone. Regarding marital status, most respondents were married or cohabited (78.8%). Regarding educational attainment, more than half of the participants (61.0%) attended primary school or below. Participants who did not smoke or drink accounted for 79.0 and 80.3%, respectively. In addition, most participants were covered by a basic medical insurance system for urban employees (44.6%) and basic endowment insurance for the urban working group (46.2%).

**Table 1:** Descriptive analysis results of participants’ characteristics (*N* = 4,218).

**Variables**	**Variable categories**	
**Age (years)**		71.9 ± 7.2
**Gender**	Male	1,484 (35.2)
	Female	2,734 (64.8)
**BMI (kg/m** ^**2**^ **)**		23.78 ± 3.50
**Residence**	Urban	2,316 (54.9)
	Rural	1902 (45.1)
**Living status**	Living alone	568 (13.5)
	Living with others	3,650 (86.5)
**Marital status**	Married	3,325 (78.8)
	single	893 (21.2)
**Education**	Primary school and below	2,571 (61.0)
	Junior school	937 (22.2)
	High school	538 (12.8)
	College and above	172 (4.1)
**Smoking status**	Smoking-quitter	307 (7.3)
	Smoker	579 (13.7)
	Non-smoker	3,332 (79.0)
**Drinking status**	Drinking-quitter	190 (4.5)
	Drinker	642 (15.2)
	Non-drinker	3,386 (80.3)
**Income**	Pension	2,344 (55.6)
	Salary	390 (9.2)
	Family provision	844 (20.0)
	Subsidy	460 (10.9)
	Others	180 (4.3)
**Medical insurance**	None	79 (1.9)
	Basic medical insurance system for urban employees	1881 (44.6)
	Basic medical insurance for urban residents	849 (20.1)
	New rural basic medical insurance for rural residents	1,399 (33.2)
	Commercial medical insurance	7 (0.2)
	Others	3 (0.1)
**Endowment insurance**	None	538 (12.8)
	Basic endowment insurance for the urban working group	1947 (46.2)
	Pension insurance for flexible employees	13 (0.3)
	Social endowment insurance for non-working urban residents	591 (14.0)
	New rural social endowment insurance for rural residents	1,110 (26.3)
	Commercial endowment insurance	19 (0.5)
**Social capital**	Social participation	9.16 ± 4.20
	Social support	13.81 ± 4.50
	Social connection	20.63 ± 3.85
	Trust	17.57 ± 3.00
	Cohesion	21.59 ± 3.51
	Reciprocity	15.69 ± 4.00
**eHealth literacy**		12.57 ± 10.00

Continuous variables are presented as range and mean ± standard deviation, categorical variables are presented as number (%).

### Correlations analysis of social capital with eHealth literacy

The Pearson’s r correlation results are presented in Table 2. Positive and significant correlations were observed (*p* < 0.05). The correlation coefficients are as follows: social participation (*r* = 0.150), social support (*r* = 0.039), social connection (*r* = 0.143), trust (*r* = 0.115), cohesion (*r* = 0.139), and reciprocity (*r* = 0.194).

**Table 2:** Correlation of included variables and eHealth literacy.

**Variables**	**1**	**2**	**3**	**4**	**5**	**6**	**7**
**1. Social participation**	1						
**2. Social support**	0.219^**^	1					
**3. Social connection**	0.067^**^	0.302^**^	1				
**4. Trust**	−0.041^**^	0.177^**^	0.625^**^	1			
**5. Cohesion**	0.049^**^	0.173^**^	0.523^**^	0.588^**^	1		
**6. Reciprocity**	0.068^**^	0.219^**^	0.392^**^	0.428^**^	0.411^**^	1	
**7. eHealth literacy**	0.150^**^	0.039^*^	0.143^**^	0.115^**^	0.139^**^	0.194^**^	1

*P<0.05, **P<0.01.

### Regression results of social capital and eHealth literacy

GLM results are presented in Tables 3–5. Table 3 displays that after controlling for confounders, attenuated but positive effects of social capital were observed (*p* < 0.05), except for social support (*p* > 0.05). Specifically, among all participants, for each unit increase in social capital as to social participation, social connection, trust, cohesion, and reciprocity, eHealth literacy scores increased by 0.15, 0.27, 0.37, 0.36, and 0.34, respectively.

**Table 3:** The relationship between social capital and eHealth literacy using GLM (all participants).

**Social capital dimensions**	**Unadjusted**		**Adjusted**	
	*B* **(SE)**	**95% CI**	*B* **(SE)**	**95% CI**
Social participation	0.36 (0.04)***	0.28–0.43	0.15 (0.04)***	0.07–0.22
Social support	0.09 (0.03)**	0.02–0.15	0.04 (0.03)	−0.02-0.10
Social connection	0.37 (0.04)***	0.29–0.45	0.27 (0.04)***	0.19–0.34
Trust	0.38 (0.05)***	0.28–0.48	0.37 (0.05)***	0.28–0.47
Cohesion	0.40 (0.04)***	0.31–0.48	0.36 (0.04)***	0.28–0.44
Reciprocity	0.48 (0.04)***	0.41–0.56	0.34 (0.04)***	0.27–0.41

Adjusted by age, gender, BMI, residency, living status, marital status, education, smoking, and drinking status, income, medical insurance, endowment insurance. B, regression coefficient; SE, standard error; 95% CI, confidence interval of 95%.

**P < 0.01,***P < 0.001.

**Table 4:** The relationship between social capital and eHealth literacy among different age groups.

**Social capital dimensions**	**60–69 years**		**70–79 years**		**≥80 years**	
	*B* **(SE)**	**95% CI**	*B* **(SE)**	**95% CI**	*B* **(SE)**	**95% CI**
Social participation	0.06 (0.06)	−0.06-0.18	1.58 (0.06)***	0.19–0.41	0.02 (0.08)	−0.13–0.17
Social support	0.02 (0.05)	−0.06-0.18	0.07 (0.05)	−0.02-0.17	0.06 (0.07)	−0.07–0.19
Social connection	0.30 (0.06)***	0.18–0.43	0.30 (0.05)***	0.20–0.41	0.13 (0.07)	−0.02–0.27
Trust	0.45 (0.08)***	0.29–0.61	0.42 (0.07)***	0.28–0.56	0.21 (0.10)*	0.02–0.40
Cohesion	0.44 (0.07)***	0.30–0.58	0.38 (0.06)***	0.26–0.50	0.23 (0.08)**	0.06–0.39
Reciprocity	0.46 (0.07)***	0.33–0.59	0.34 (0.05)***	0.24–0.44	0.19 (0.06)**	0.07–0.32

Adjusted by, BMI, gender, residency, living status, marital status, education, smoking, and drinking status, income, medical insurance, endowment insurance. B, regression coefficient; SE, standard error; 95% CI, confidence interval of 95%.

*P < 0.05,**P < 0.01,***P < 0.001.

**Table 5:** The relationship between social capital and eHealth literacy among different genders.

**Social capital dimensions**	**Male**		**Female**	
	*B* **(SE)**	**95% CI**	*B* **(SE)**	**95% CI**
Social participation	0.14 (0.06)*	0.02–0.26	0.15 (0.05)**	0.06–0.24
Social support	0.00 (0.05)	−0.11–0.11	0.06 (0.04)	−0.01-0.14
Social connection	0.16 (0.07)*	0.03–0.29	0.34 (0.05)***	0.25–0.43
Trust	0.33 (0.08)***	0.16–0.50	0.39 (0.06)***	0.28–0.51
Cohesion	0.32 (0.07)***	0.18–0.45	0.39 (0.05)***	0.29–0.49
Reciprocity	0.41 (0.06)***	0.28–0.54	0.31 (0.04)***	0.23–0.39

Adjusted by age, BMI, residence, living status, marital status, education, smoking, and drinking status, income, medical insurance, endowment insurance. B, regression coefficient; SE, standard error; 95% CI, confidence interval of 95%. *P < 0.05, **P < 0.01,***P < 0.001.

In Table 4, we found that age differences existed when social capital was linked to eHealth literacy in different age groups. Specifically, trust, cohesion, and reciprocity were positively and significantly linked to eHealth literacy among participants from the three age groups (*p* < 0.05). Meanwhile, social connection was positively and significantly linked to eHealth literacy for participants aged 60–69 and 70–79 years (*p* < 0.001). Finally, social participation was only linked to eHealth literacy among participants aged 70–79 years after adjustment (*p* < 0.001).

However, no statistically significant gender differences were identified (Table 5). In stratified analysis by gender, the social capital associated with eHealth literacy scores was the same among male and female respondents, while this association was stronger in females than in males.

## Discussion

This study examined the relationship between social capital and eHealth literacy in four cities in the Yangtze River Delta region, China. Overall, the significant positive association of social capital dimensions with eHealth literacy scores was identified based on our data analyses. In other words, community-dwelling older people with higher social capital concerning social participation, social connection, trust, cohesion, and reciprocity reported a better eHealth literacy status. In addition, age differences were observed when linking social capital to eHealth literacy, whereas such differences did not exist when considering gender.

Similar to previous studies, we observed that after adjustment for confounding factors in the total population, a higher level of eHealth literacy could be found when high-ranking social capital scores concerning social connection, social participation, trust, cohesion, and reciprocity appeared (23–25). For example, the role of social participation, social support, and social connection on eHealth literacy has been proven in a previous study (23). Social connection can enhance social interaction and supplement eHealth literacy (25). Moreover, evidence has documented that higher cognitive social capital grades (trust, cohesion, and reciprocity) are conducive to obtaining redundant information in a network; therefore, acquiring knowledge and higher health awareness could eventually enhance eHealth literacy (8, 39). However, such findings were inconclusive, and no positive correlation between cognitive social capital and eHealth literacy was obtained in a previous study (23), which is inconsistent with our findings. To understand this, it is highly likely that different tools were used to assess social capital. Specifically, in the above-mentioned study, the subscale consisted of trust, cohesion, and reciprocity, a total of 11 items, and was used to assess cognitive social capital (23), indicating that more research is needed in the future.

Previous studies have depicted that perceived social support and informational social support contribute to the acquisition of eHealth knowledge and growth of eHealth literacy (40, 41), different from our findings. This may be because of the different scales for measuring social support. For example, a multidimensional scale consisting of 12 items was used to measure perceived social support, which measured two sources of support (family and friends) (40). Informational social support includes four sub-concepts: emotional support, informational support that provides information that can be used to address personal problems, material support (monetary or material help), and evaluative support (acknowledgment or respect) (41). This study used four items to assess social capital (mental and material support).

Moreover, age differences were observed when social capital was associated with eHealth literacy based on our analyses. In particular, social capital regarding trust, cohesion, and reciprocity was positively and significantly linked to eHealth literacy scores among participants of all ages in this study. One possible explanation for this finding could be that high levels of trust, reciprocity, and cohesion are positively correlated with optimism among older people, making them believe in electronic programs and online information, which may make them more likely to accept eHealth information (42). Meanwhile, a previous study demonstrated the importance of the quality of trust, cohesion, and reciprocity in determining the subjective well-being and self-assessed health status of older people in later life, increasing the availability of healthy social support resources (43, 44). In addition, self-rated health was associated with eHealth literacy, which may imply that older people with better health literacy provide more possibilities for further improving eHealth literacy (45).

In addition, social capital concerning social connection was positively and significantly linked to eHealth literacy scores, especially among participants aged 60–69 and 70–79 years. This echoed a study finding that a higher level of social connection could improve cognitive function and memory and that better cognitive ability may lead to increased access to the Internet and adequate health literacy, which may help older people better seek eHealth help (46).

More interestingly, our study found that social participation, a social capital dimension, was only linked to eHealth literacy among participants aged 70–79 years, which is similar to results in a previous study from Ghana (47) indicating that active participation in association activities can enhance health promotion choices by promoting access to important health-related information.

Based on our study results, gender differences did not exist. This is incompatible with the findings in previous studies (28), noting that older female immigrants were less capable of accessing the Internet to find useful health resources than their male counterparts and had fewer skills with which to access health resources, thereby resulting in a relative lack of eHealth literacy. Meanwhile, males were found to have a higher level of eHealth literacy than females among college students in a previous study (48).

Similar conclusions have been previously reached. A study on left-behind older adults in rural China demonstrated that, in comparison with older males, the role of social capital in mental health preservation was more significant for older females (49). A possible reason for this result is that females have a high degree of social trust, prefer to make friends, and are more likely to seek support from others and mobilize support resources (especially emotional support). Therefore, females have a higher network density; accordingly, some social capital indicators have a more significant protective effect on females (49, 50).

These mixed association models could inform programs to promote the eHealth literacy of older people from a social capital perspective. Moreover, when linking social capital to eHealth literacy, age and gender should be fully considered. Therefore, based on our findings, the following suggestions can be provided: First, we recommend that older people be encouraged and supported in their efforts to participate in social activities, with special attention given to the role of family members. Children and relatives should be encouraged to strengthen social support for older people and improve accessibility to electronic devices to collect and discern health information. Second, with the opportunity to vigorously promote the policy of building age-friendly communities in China (51), the community should effectively undertake health management measures and enhance interpersonal communication and mutual learning among older people to facilitate the exchange of health information, increase social participation, and encourage older people to abide by reciprocal norms and increase their sense of trust. Third, enterprises and primary healthcare institutions should attach importance to a sense of social responsibility. Electronic products should be designed to be suitable for older people or provide electronic health services. Finally, the government should provide policy support to reduce this digital divide. For example, financial subsidies should be provided, and Internet coverage should be improved. However, relevant social organizations should be coordinated and organized to offer multimedia tutorial courses to improve eHealth literacy, such as nursing homes and universities for older people (52), which is in line with the Chinese government’s strategic policy of actively promoting smart healthcare (53).

Nevertheless, we acknowledge that this study has a few limitations. First, a cross-sectional study was used, which might limit the conclusion of the causal relationship between social capital and eHealth literacy. Further research should use longitudinal data and a randomized controlled trial design. On the other hand, data on social capital were only obtained by individual-level measurement, and whether community-level social capital has the same relationship with eHealth literacy needs further explored. Such an examination may help widen the application of social capital. Despite the aforementioned limitations, we believe that using a large representative sample with a good response rate and the employment of valid and reliable measurement tools to collect data could be interpreted as the strengths of this study.

## Conclusion

In conclusion, a correlation between social capital and eHealth literacy was identified based on our data analyses among older people living in a community setting. Specifically, older people with high levels of social connection, social participation, trust, reciprocity, and cohesion are more likely to prefer eHealth literacy in later life. Furthermore, this association varies with age. Our research could inform the development of personalized strategies from a social capital perspective to improve eHealth literacy among older people, which is essential for achieving active and healthy aging.

## Data availability statement

The raw data supporting the conclusions of this article will be made available by the authors, without undue reservation.

## Ethics statement

The studies involving human participants were reviewed and approved by the Ethics Committee of Anhui Medical University. The patients/participants provided their written informed consent to participate in this study.

## Author contributions

CC contributed to the design and writing of the article. WC, XZ, KJ, and YW contributed to data analyses. ZH, RC, and ZB contributed to funding acquisition, quality control, and data processing and revised the manuscript. All authors have read and approved the final version of the manuscript.

## Funding

This work was supported by the China Scholarship Council (no. 202209095002), National Natural Science Foundation of China (no. 71573002), Special Research Project in Science and Technology Department of Anhui Province (no. 202106f01050045), Key Project of Social Science in Education Department of Anhui Province (no. SK2021A0164), Research Fund of Anhui Medical University (no. 2021xkjT049), and Open Program of Health Policy Research Center of Anhui Medical University (no. 2022wszc19).

## Conflict of interest

The authors declare that the research was conducted without commercial or financial relationships that could be construed as potential conflicts of interest.

## Publisher’s note

All claims expressed in this article are solely those of the authors and do not necessarily represent those of their affiliated organizations, or those of the publisher, the editors and the reviewers. Any product that may be evaluated in this article, or claim that may be made by its manufacturer, is not guaranteed or endorsed by the publisher.
